# Scintigraphic Findings in FGF23-related Hypophosphatemic Osteomalacia

**DOI:** 10.31662/jmaj.2022-0223

**Published:** 2023-03-24

**Authors:** Naoya Fujita, Yosuke Ono

**Affiliations:** 1 Department of General Medicine, National Defense Medical College, Tokorozawa, Japan

**Keywords:** FGF23-related hypophosphatemic osteomalacia, bone scintigraphy, tie sign, rachitic rosary sign

A 67-year-old woman presented with systemic bone pain and muscle weakness. Laboratory examinations revealed high levels of serum alkaline phosphatase, normal corrected calcium, and low inorganic phosphate levels. Bone scintigraphy revealed multiple uptake in the sternum, costochondral junction, and knee joints, which were known as the “tie sign,” “rachitic rosary sign,” and “accumulation in the knees,” respectively (Figure 1a) ^(1), (2), (3)^. These signs are the characteristic of fibroblast growth factor 23 (FGF23)-related hypophosphatemic osteomalacia, and the serum FGF23 levels were high (184 pg/mL; normal range: 19.9-52.9 pg/mL). Although she was suspected to have tumor-induced osteomalacia, systemic venous samplings of FGF23 and ^111^In-octreotide somatostatin receptor scintigraphy were unable to identify the tumor. Her symptoms and multiple uptake on bone scintigraphy almost disappeared after initiating active vitamin D and phosphate replacement (Figure 1b). Although bone scintigraphy is not useful for localizing tumors, it is recommended to differentiate the etiology of bone pain.

**Figure 1.**
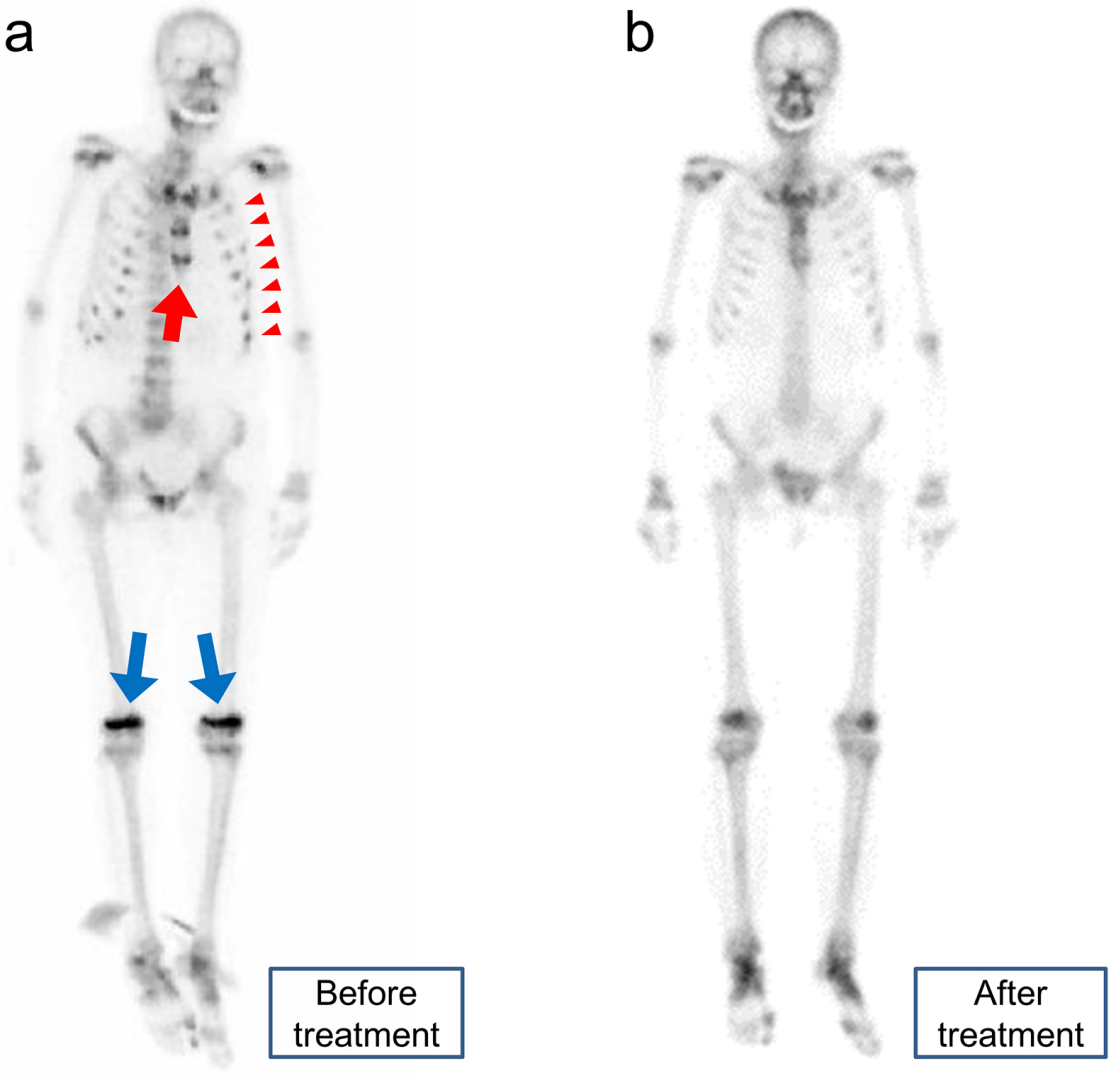
a. Bone scintigraphy before treatment. Multiple foci of increased tracer uptake were observed in the sternum (red arrow), costochondral junction (red arrowheads), and knee joints (blue arrows). These are known as the “tie sign,” “rachitic rosary sign,” and “accumulation in the knees,” respectively. b. Bone scintigraphy after treatment. The multiple uptake sites have almost disappeared.

## Article Information

### Conflicts of Interest

None

### Author Contributions

 Naoya Fujita wrote the first draft. Yosuke Ono suggested improvements and revised the manuscript.

### Informed Consent

Written informed consent was obtained from the patient to publish this report in accordance with the journal’s patient consent policy.
